# Factors Associated with Failed Treatment: an Analysis of 121,744 Women Embarking on Their First IVF Cycles

**DOI:** 10.1371/journal.pone.0082249

**Published:** 2013-12-05

**Authors:** Siladitya Bhattacharya, Abha Maheshwari, Jill Mollison

**Affiliations:** Division of Applied Health Sciences, University of Aberdeen, Aberdeen, United Kingdom; University of Louisville, United States of America

## Abstract

**Background:**

In-vitro fertilization (IVF) is the treatment of choice for unresolved infertility. It comprises a number of key steps, each of which has to be negotiated before the next is attempted, but the factors which are associated with failure at each stage have not been reported.

**Methods and Findings:**

We analyzed anonymised national data on women undergoing their first fresh autologous IVF and intracytoplasmic sperm injection (ICSI) cycle in the United Kingdom between 2000 and 2007 to predict factors associated with overall lack of livebirth as well as the chance of non-progress at different stages of an IVF cycle. A total of 121,744 women were included in this analysis. Multivariable models underlined the importance of increased female age and duration of infertility, lack of previous pregnancy, and a diagnosis of tubal or male factor infertility in predicting the risk of not having a live birth in an IVF treatment. At each stage, a woman’s chance of proceeding to the next stage of IVF treatment is affected by increased age and duration of infertility. The intention to use intra-cytoplasmic sperm injection (ICSI) is associated with a decreased risk of treatment failure in women starting an IVF cycle (RR 0.93, 99% CI 0.92, 0.94) but this association is reversed at a later stage once fertilisation has been confirmed (RR=1.01, 99%CI 1.00, 1.03).

**Conclusions:**

Female age is a key predictor of failure to have a livebirth following IVF as well as the risk of poor performance at each stage of treatment. While increased duration of infertility is also associated with worse outcomes at every stage, its impact appears to be less influential. Women embarking on ICSI treatment for male factor infertility have a lower chance of treatment failure but this does not appear to be due to increased chances of implantation of ICSI embryos.

## Introduction

In-vitro fertilization (IVF) is the recommended treatment of choice for unresolved infertility (NCCWCH NICE, 2013). In 2011, over 48,147 women in the United Kingdom underwent a total of 61,726 cycles of IVF or intracytoplasmic sperm injection (ICSI) (HFEAhttp://www.hfea.gov.uk). 

A number of studies in the literature have explored the impact of factors predicting the successful outcome of IVF in terms of pregnancy and/or livebirth. Most have relied on analyses of routinely collected data based on cycles of treatment and the number of co-variates have been in inverse proportion to the size of the dataset [1-17]. While factors depending on ovarian function such as female age and number of oocytes as well as duration of infertility which is also associated with female age have been identified as predictors of pregnancy, a systematic review of studies called for further research in this area[17].

The Human Fertilisation and Embryology Authority (HFEA) has collected data on all licensed fertility treatments in the UK since 1991. Analysis of IVF and ICSI cycles from different time periods within this dataset by Templeton[18], and Nelson [15] has identified predictors of livebirth following IVF. Both studies identified female age, duration of infertility and previous pregnancy as key prognostic factors. In addition, Nelson and Lawlor, working with a larger, more recent dataset including ICSI as well as IVF cycles, found cause of infertility and the use of ICSI to be additional factors associated with successful treatment.

Both studies used cycles, rather than individual women as their unit of analysis, and focused on a global prediction of live birth. IVF comprises a number of key stages - including controlled ovarian stimulation oocyte retrieval, fertilisation, embryo transfer and confirmation of early pregnancy - each of which has to be negotiated before the next can be attempted. With a reported U.K. national live birth rates of 32% (HFEAhttp://www.hfea.gov.uk) in women aged 18-34 years, the majority of IVF attempts are not successful. Couples and clinicians are interested in understanding reasons for treatment failure and the role of clinical characteristics associated with non-progress to the next stage but none of the studies in the literature has addressed this. Additionally, recent years have seen the emergence of term singleton live birth[19] as a preferred outcome of IVF, but one which is infrequently reported by existing prognostic models. 

The HFEA data set remains a rich source of material for investigating factors influencing the overall outcome of IVF as well as at each stage. In this study, using HFEA data from women in their first IVF cycles, we aimed to determine the chance of treatment failure (defined as absence of livebirth and term singleton livebirth) in women attempting IVF for the first time and to determine factors associated with the inability to progress on to the next stage of IVF. We also investigated the changes in the risk of unsuccessful treatment at each stage of treatment.

We analyzed anonymised HFEA data (http://www.hfea.gov.uk) from 2000 to 2007 on women undergoing their first fresh IVF and ICSI cycles using their own eggs. We estimated factors associated with global treatment failure as well as poor outcomes at each stage of an IVF cycle. 

## Methods

### Patients

**Figure 1:**
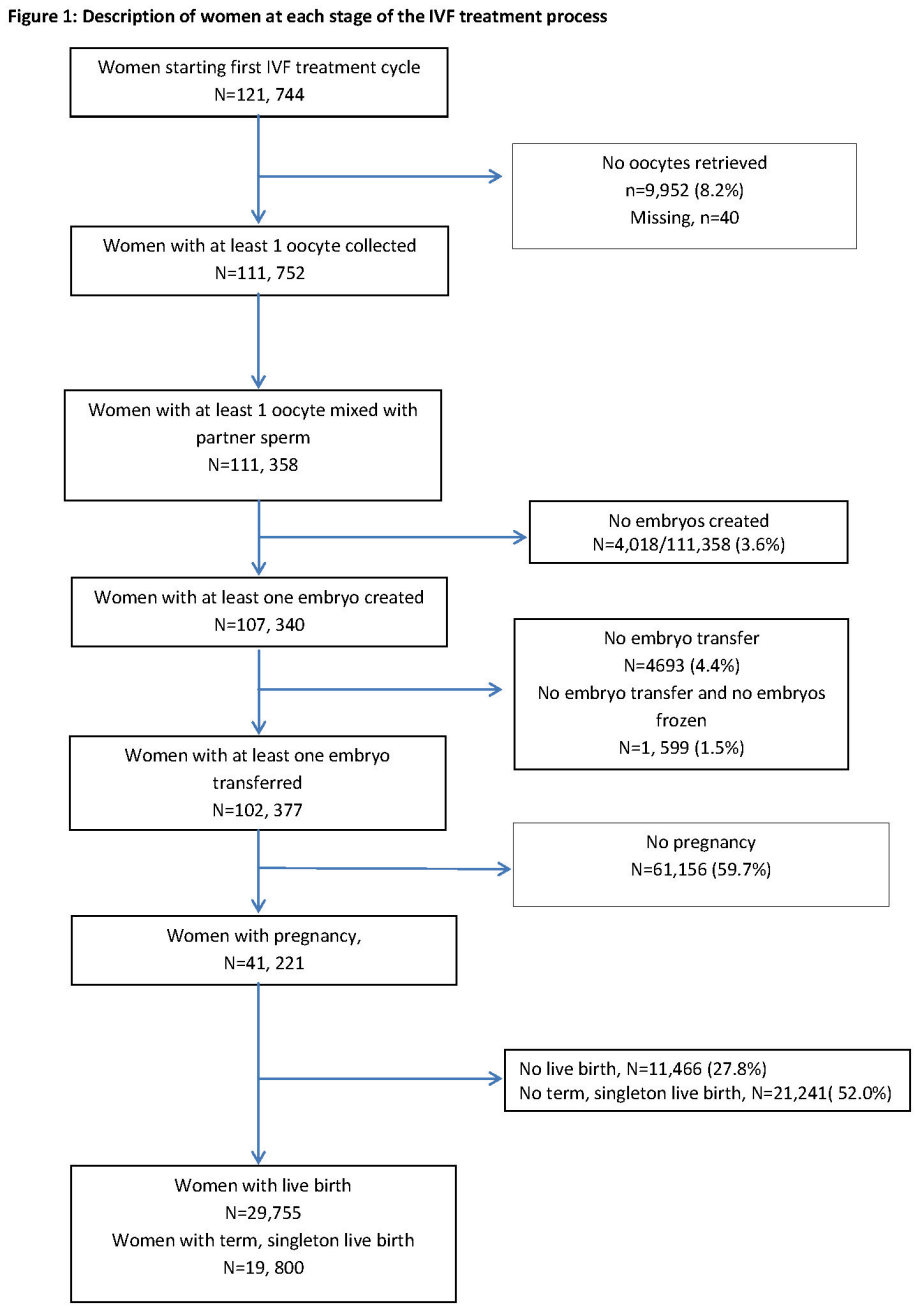
Description of women at each stage of the IVF treatment process.

We utilised anonymised data from the HFEA register and included all cycles where women were undergoing their first, fresh, autologous IVF and ICSI cycles between January 2000 and December 2007. We excluded cycles involving egg donation or those initiated with the express purpose of storage of eggs/embryos rather than fresh embryo transfer. (Figure 1). Restricting the analysis to first cycles enabled us to report rates of failure per individual woman.

### Outcomes

Poor ovarian response was defined as retrieval of three or less oocytes per woman[20] . In women who had eggs mixed or injected with sperm, poor fertilisation was defined as fertilisation of fewer than 20% of oocytes. In women who reached embryo transfer, failure to achieve pregnancy was defined as a negative pregnancy test. Live birth was defined as at least one baby born alive and a term singleton live birth was defined as a single baby born at or beyond 37 weeks gestation. 

### Covariates

Duration and cause of infertility were categorised in accordance with previous analyses of HFEA data[15]. Previous pregnancy related to whether the women had primary infertility or not as recorded in HFEA database. For numbers of embryos transferred, we combined 3 and 4 embryo transfers into a single category and the reference category was double embryo transfer. The number of fresh oocytes retrieved was categorised into the following groups; 1-4 oocytes, 5-9 oocytes, 10-14 oocytes (reference category), 15-19 oocytes and 20 or more oocytes. Our choice of categories was informed by previous work using HFEA data and we choose 10 -14 oocytes as the reference category based on the assumption that an optimal outcome could be anticipated in this group[21]. To reflect embryo quality we derived a variable- embryo utilisation, defined as the proportion of all embryos created that were either transferred or frozen. We did not include elective single embryo transfer (eSET) as a covariate due to very few cases during this time period, only 63 (0.1%) first cycles during this period involved eSET. 

### Statistical analysis

We performed an analysis using data from all women embarking on their first cycles to assess associations with failure to achieve a live birth. We examined whether the association between prognostic factors and failure to achieve live birth changed over the course of treatment, by fitting the same model to the sample of women who achieved each stage in the course of treatment. The analysis at each stage in the IVF process was based on only those women who had reached the preceding stage. For example, in the analysis of ovarian response, all women were included; for fertilisation, the denominator was all women in whom at least one oocyte was mixed with sperm, for embryo transfer the denominator was all women where at least one embryo was created, and for positive pregnancy test the denominator was all women who had at least one embryo transferred. By utilising a prospectively collected database and clearly defining our cohort for every analyses, we could estimate the risk that a women would experience each specific outcome.

We examined likelihood of failure and factors associated with failure overall and at each stage in the IVF cycle using univariable and multivariable Poisson regressions with robust error variance[22,23]. Poisson regression was preferred over logistic regression since many of our outcomes were common (e.g. failure of pregnancy with fertility treatment (~75%)) and the odds ratio is known to overstate the risk ratio in these circumstances[24]. Risk ratios in multivariable models were adjusted for all other variables in the model. For the outcomes of fertilisation and positive pregnancy test (2 weeks after embryo transfer) we stratified the analysis by the method of fertilisation i.e. IVF or ICSI. In all other analyses, treatment by IVF or ICSI was included as a covariate. All statistical analyses were performed using IBM SPSS version 20 (SPSS Inc.). Age of women at treatment, duration of infertility, number of oocytes retrieved, number of embryos transferred, embryo utilisation and outcome were fitted as categorical variables in the regressions. The extent of missing data was documented. In multivariable regressions any cycles with incomplete data on outcome or covariates were excluded from the analysis. As the anonymised data are freely available on the HFEA website, formal ethics approval was not required for this analysis.

## Results

Data from 121,744 women undergoing their first cycle of IVF or ICSI were included in this analysis (Figure 1). Of these, 72,410 women received IVF and 49,334 had ICSI. 

### Live birth

Of women undergoing their first IVF or ICSI cycles 91,749 (75.4%) did not have a live birth. The factors associated with an overall unsuccessful treatment outcome were older age, increasing duration of infertility, no previous pregnancy and treatment by IVF (Table 1). After adjustment for all other variables, women were more likely to not have a live birth when the diagnosis was tubal disease, male factor or a combination of known factors, in comparison with cases where the cause of infertility was unexplained. When we examined how the relationship between these factors and failure to achieve live birth changed over the course of treatment, the impact of age, duration of infertility and previous pregnancy remained reasonably constant at all stages (Table 2). However, with regards to fertilisation by IVF or ICSI, the association with unsuccessful treatment changed with the stage of treatment. When all women embarking on IVF or ICSI were considered, the intention to use ICSI was associated with a decreased risk of failure (RR 0.93, 99% CI 0.92, 0.94), but following successful oocyte retrieval, the insemination method was no longer independently associated with live birth (RR 1.0, 99% CI 0.98, 1.0). In fact, when only women who reached the stage of fertilisation (embryo creation) or embryo transfer were included in the analysis, treatment by ICSI was independently associated with a slight increase in risk of failure to achieve live birth. The relationship between cause of infertility and unsuccessful treatment outcome also changed over the course of treatment. At the start of a treatment cycle, couples with a diagnosis of male factor only were at an increased chance of failure (RR 1.05 99% CI 1.03, 1.06) compared to couples with unexplained infertility (used as the reference category) but this disappeared at the next stage as women progressed through the IVF cycle (RR 1.0. 99% CI 0.99, 1.02). The impact of tubal infertility on the risk of treatment failure continued to grow at each stage. Poor embryo utilisation (<50% of all embryos created either transferred or frozen) increased women’s chance of non-live birth when only women with at least one embryo transferred were considered. 

**Table 1:** Factors associated with non-live birth in all women starting their first cycle of IVF treatment.

	**Number of women in each category**	**Percentage within each category with no live birth**	**Univariable risk ratio of no live birth (99% CI)**	**P value**	**Multivariable risk ratio of no live birth (99% CI)**	**P value^*2*^**
			**N=121, 744 women included in analysis**		**N=108, 889 women included in analysis^*1*^**	
**All women**	**121, 744**	**91,749 (75.4%)**				
**Women’s age (years)**				P<0.001		P<0.001
18-34	63685	70.3%	1.0		1.0	
35-37	28867	75.2%	1.07 (1.06, 1.08)		1.07 (1.06, 1.08)	
38-39	14853	81.6%	1.16 (1.15, 1.17)		1.17 (1.15, 1.18)	
40-42	11140	90.0%	1.28 (1.27, 1.29)		1.29 (1.27, 1.30)	
43-44	2435	97.0%	1.38 (1.37 , 1.40)		1.39 (1.37, 1.41)	
45-50	764	98.2%	1.40 (1.38, 1.42)		1.41 (1.39, 1.43)	
Missing	0					
**Duration (years)**				P<0.001		P<0.001
<1	2017	67.8%	0.90 (0.86, 0.94)		0.89 (0.85, 0.92)	
1-3	45799	73.5%	0.98 (0.97, 0.99)		0.96 (0.95, 0.97)	
4-6	38029	75.4%	1.0		1.0	
7-9	12835	77.7%	1.03 (1.02, 1.05)		1.03 (1.01, 1.04)	
10-12	6762	78.9%	1.05 (1.03, 1.07)		1.03 (1.01, 1.05)	
>12	6529	81.8%	1.09 (1.07, 1.10)		1.04 (1.02, 1.06)	
Missing	9773 (8.0%)					
**Cause of infertility**				P<0.001		P<0.001
Tubal disease only	20141	77.5%	1.03 (1.02, 1.04)		1.05 (1.04, 1.07)	
Ovulatory only	8779	77.0%	1.02 (1.01, 1.04)		1.02 (1.00, 1.04)	
Male Factor Only	37448	73.4%	0.98 (1.96, 0.99)		1.05 (1.03, 1.06)	
Unexplained	32629	75.3%	1.0		1.0	
Endometriosis	4097	73.3%	0.97 (0.95, 1.00)		1.0 (0.97, 1.02)	
Cervical Factors only	52	88.5	1.17 (1.03, 1.34)		1.16 (1.01, 1.34)	
Combination of Known factors	14268	76.1%	1.01 (1.00, 1.03)		1.06 (0.04, 1.07)	
Missing	4330 (3.6%)					
**Previous pregnancy**				P=0.001		P<0.001
Yes	71683	75.9%	1.0		1.0	
No	50061	75.0%	0.99 (0.98, 1.00)		1.04 (1.03, 1.05)	
**Treatment**				P<0.001		P<0.001
IVF	72410	77.5%	1.0		1.0	
ICSI	49334	72.3%	0.93 (0.93, 0.94)		0.93 (0.92, 0.94)	

^1^ Multivariable risk ratio is adjusted for all variables listed in the table

^2^ P values obtained from multivariable model.

**Table 2:** Factors associated with no live birth in women achieving each stage in IVF process.

	**All women starting 1^st^ cycle**		**Women who had at least 1 oocyte retrieved**		**Women with at least 1 embryo created**		**Women with at least 1 embryo transferred**		**Women who had positive pregnancy test**	
	**Univariable RR^1^ N=121,744**	**Multivariable RR (99% CI) N=108,889**	**Univariable RR N=111, 752**	**Multivariable RR (99% CI) N=100, 091**	**Univariable RR N=107, 340**	**Multivariable RR (99% CI) N=96, 227**	**Univariable RR N=102, 377**	**Multivariable RR (99% CI) N=91, 903**	**Univariable RR N=41,221**	**Multivariable RR (99%CI) N=37,018**
**All women n(%)**	**91,749 (75.4%)**	**81,929 (75.2%)**	**81,769 (73.2%)**	**73,142 (73.1%**	**77,370(72.1%)**	**69,291 (72.0%)**	**72,438 (70.8%)**	**64, 997 (70.7%)**	**11,466 (27.8%)**	**10, 276 (27.8%)**
**Women’s age (years)**										
18-34	1.0	1.0	1.0	1.0	1.0	1.0	1.0	1.0	1.0	1.0
35-37	1.07 (1.06, 1.08)	1.07 (1.06, 1.08)	1.07 (1.06, 1.08)	1.07 (1.06, 1.09)	1.07 (1.06, 1.09)	1.07 (1.05, 1.08)	1.09 (1.07, 1.10)	1.07 (1.06, 1.09)	1.15 (1.09, 1.21)	1.14 (1.08, 1.21)
38-39	1.16 (1.15, 1.17)	1.17 (1.15, 1.18)	1.17 (1.15, 1.18)	1.18 (1.16, 1.19)	1.17 (1.15, 1.19)	1.16 (1.14, 1.17)	1.19 (1.17, 1.21)	1.16 (1.15, 1.18)	1.43 (1.34, 1.52)	1.43 (1.34, 1.52)
40-42	1.28 (1.27, 1.29)	1.29 (1.27, 1.30)	1.30 (1.28, 1.31)	1.31 (1.29, 1.33)	1.31 (1.29, 1.33)	1.28 (1.26, 1.30)	1.34 (1.32, 1.36)	1.28 (1.26, 1.30)	1.91 (1.79, 2.04)	1.83 (1.69, 1.97)
43-44	1.38 (1.37, 1.40)	1.39 (1.37, 1.41)	1.42 (1.40, 1.43)	1.43 (1.41, 1.45)	1.43 (1.41,1.45)	1.38 (1.35, 1.40)	1.47 (1.45, 1.49)	1.37 (1.35, 1.40)	2.68 (2.35, 3.06)	2.39 (2.04, 2.80)
45-50	1.40 (1.38, 1.42)	1.41 (1.39, 1.43)	1.43 (1.41, 1.46)	1.46 (1.43, 1.49)	1.45 (1.43, 1.48)	1.39 (1.36, 1.42)	1.49 (1.46, 1.52)	1.38 (1.34, 1.41)	2.58 (1.87, 3.55)	2.50 (1.83, 3.43)
**Duration (years)**										
<1	0.90 (0.86, 0.94)	0.89 (0.85, 0.92)	0.90 (0.86, 0.94)	0.88 (0.85, 0.92)	0.89 (0.85, 0.94)	0.88 (0.84, 0.92)	0.88 (0.84, 0.93)	0.87 (0.83, 0.92)	1.06 (0.92, 1.22)	1.02 (0.88, 1.17)
1-3	0.98 (0.97, 0.99)	0.96 (0.95, 0.97)	0.98 (0.96, 0.99)	0.96 (0.95, 0.97)	0.98 (0.97, 0.99)	0.96 (0.95, 0.98)	0.98 (0.97,0.99)	0.96 (0.95, 0.98)	0.98 (0.93, 1.03)	0.95 (0.90, 1.00)
4-6	1.0	1.0	1.0	1.0	1.0	1.0	1.0	1.0	1.0	1.0
7-9	1.03 (1.02, 1.05)	1.03 (1.01, 1.04)	1.03 (1.02, 1.05)	1.03 (1.01, 1.04)	1.03 (1.01, 1.05)	1.02 (1.01, 1.04)	1.03 (1.01, 1.05)	1.02 (1.0, 1.04)	1.10 (1.02, 1.18)	1.06 (0.99, 1.14)
10-12	1.05 (1.03, 1.07)	1.02 (1.01, 1.05)	1.05 (1.02, 1.07)	1.03 (1.01, 1.05)	1.05 (1.02, 1.07)	1.02 (1.00, 1.04)	1.05 (1.03, 1.07)	1.02 (1.00, 1.05)	1.15 (1.05, 1.26)	1.09 (0.99, 1.19)
>12	1.09 (1.07, 1.10)	1.04 (1.02, 1.06)	1.09 (1.07, 1.11)	1.04 (1.02, 1.06)	1.09 (1.07, 1.11)	1.03 (1.01, 1.06)	1.10 (1.08, 1.12)	1.04 (1.02, 1.06)	1.24 (1.13, 1.36)	1.09 (0.98, 1.20)
**Cause of infertility**										
Tubal disease only	1.03 (1.02, 1.04)	1.05 (1.04, 1.07)	1.03 (1.02, 1.05)	1.06 (1.05, 1.08)	1.04 (1.02, 1.06)	1.07 (1.06, 1.09)	1.04 (1.03, 1.06)	1.08 (1.06, 1.10)	1.09 (1.03, 1.16)	1.15 (1.08, 1.17)
Ovulatory only	1.02 (1.01, 1.04)	1.02 (1.00, 1.04)	1.01 (0.99, 1.03)	1.01 (0.99, 1.03)	1.01 (0.99, 1.03)	1.01 (0.99, 1.03)	0.99 (0.97, 1.02)	1.00 (0.98, 1.02)	1.02 (0.94, 1.11)	1.07 (0.98, 1.17)
Male Factor Only	0.98 (1.96, 0.99)	1.05 (1.03, 1.06)	0.97 (0.97, 0.99)	1.00 (0.99, 1.02)	0.98 (0.97, 0.99)	1.00 (0.98, 1.02)	0.98 (0.97, 0.99)	1.00 (0.99, 1.02)	0.94 (0.89, 0.99)	0.98 (0.91, 1.05)
Unexplained	1.0	1.0	1.0	1.0	1.0	1.0	1.0	1.0	1.0	1.0
Endometriosis	0.97 (0.95, 1.00)	1.0 (0.97, 1.02)	0.97 (0.94, 1.0)	1.00 (0.97, 1.02)	0.97 (0.94, 1.00)	0.99 (0.96, 1.02)	0.97 (0.94, 1.00)	1.00 (0.96, 1.03)	0.92 (0.82, 1.04)	0.97 (0.86, 1.10)
Cervical Factors only	1.17 (1.03, 1.34)	1.16 (1.01, 1.34)	1.19 (1.02, 1.38)	1.19 (1.01, 1.40)	1.19 (1.01, 1.40)	1.21 (1.01, 1.45)	1.20 (0.99, 1.45)	1.20 (0.96, 1.59)	1.34 (0.41, 4.36)	1.49 (0.45, 4.96)
Combination of Known factors	1.01 (1.00, 1.03)	1.06 (0.04, 1.07)	1.01 (0.99, 1.02)	1.03 (1.01, 1.05)	1.01 (0.99, 1.03)	1.03 (1.01, 1.04)	1.01 (0.99, 1.03)	1.03 (1.01, 1.05)	1.03 (0.96, 1.11)	1.08 (1.00, 1.16)
**Previous pregnancy**										
Yes	1.0	1.0	1.0	1.0	1.0	1.0	1.0	1.0	1.0	1.0
No	0.99 (0.98, 1.00)	1.04 (1.03, 1.05)	0.99 (0.98, 1.00)	1.03 (1.02, 1.04)	0.99 (0.98, 0.99)	1.03 (1.02, 1.04)	0.98 (0.97, 0.99)	1.03 (1.02, 1.04)	0.90 (0.87, 0.94)	0.99 (0.94, 1.03)
**Treatment**										
IVF	1.0	1.0	1.0	1.0	1.0	1.0	1.0	1.0	1.0	1.0
ICSI	0.93 (0.93, 0.94)	0.93 (0.92, 0.94)	0.98 (0.97, 0.99)	1.00 (0.98. 1.01)	0.99 (0.98, 1.0)	1.01 (1.00, 1.03)	0.99 (0.98, 1.00)	1.02 (1.00, 1.03)	0.96 (0.92, 1.00)	1.04 (0.98, 1.10)
**Number of oocytes retrieved**										
1-4			1.27 (1.25, 1.28)	1.21 (1.19, 1.22)	1.25 (1.23, 1.27)	1.19 (1.17, 1.21)	1.25 (1.23, 1.27)	1.14 (1.12, 1.17)	1.29 (1.20, 1.39)	1.20 (1.11, 1.30)
5-9			1.10 (1.09, 1.12)	1.09 (1.07, 1.10)	1.10 (1.09, 1.12)	1.08 (1.07, 1.10)	1.11 (1.09, 1.12)	1.08 (1.07, 1.10)	1.13 (1.08, 1.19)	1.11 (1.05, 1.17)
10-14			1.0	1.0	1.0	1.0	1.0	1.0	1.0	1.0
15-19			0.99 (0.97, 1.00)	1.00 (0.98, 1.02)	0.99 (0.97, 1.01)	1.00 (0.98, 1.02)	0.98 (0.96, 1.0)	0.99 (0.97, 1.01)	1.02 (0.95, 1.09)	1.06 (0.99, 1.13)
20+			1.05 (1.03, 1.07)	1.07 (1.05, 1.09)	1.06 (1.04, 1.08)	1.07 (1.05, 1.09)	0.96 (0.93, 0.98)	0.97 (0.95, 1.00)	0.98 (0.91, 1.06)	1.01 (0.93, 1.10)
**Num. of embryos transferred**										
One							1.26 (1.25, 1.28)	1.17 (1.15, 1.18)	1.24 (1.14, 1.35)	1.17 (1.07, 1.28)
Two							1.0	1.0	1.0	1.0
>Three							1.14 (1.12, 1.16)	1.04 (1.03, 1.06)	1.29 (1.20, 1.39)	1.06 (0.98, 1.15)
**Embryo Utilisation^*2*^**										
0-25%							0.92 (0.91, 0.94)	1.03 (1.01, 1.05)	0.98 (0.91, 1.04)	1.05 (0.98.1.13)
25-50%							0.97 (0.96, 0.98)	1.02 (1.01, 1.04)	1.03 (0.98, 1.08)	1.05 (0.99, 1.11)
50-75%							0.94 (0.93, 0.96)	0.99 (0.98, 1.01)	0.99 (0.94, 1.05)	1.00 (0.94, 1.07)
75-100%							1.0	1.0	1.0	1.00

^1^ RR - risk ratio

***^2^*** Embryo utilisation is defined as (number of embryos transferred + number of embryos frozen)/total number of embryos created

### Ovarian response

Of all women starting their first IVF treatment cycle, 20,621 (16.9%) had 3 or fewer oocytes retrieved (Table 3). The proportion of women with few oocytes was strongly influenced by age, being lowest in 18 - 34 year olds (11.8%) and highest in those aged over 45 years (51%). The risk of low oocyte yield was also independently associated with increasing duration of infertility, no previous pregnancy and women with a diagnosis of endometriosis, male factor, or anovulation relative to women with an unexplained diagnosis. Univariately, a diagnosis of male factor infertility was associated with a reduced risk of low oocyte yield (RR 0.87, 99% CI 0.83, 0.91), possibly reflecting the fact that these women tended to be younger. This effect disappeared once female age and other factors were adjusted for in the multivariate model (RR 1.98, 99% CI 1.88, 2.09). 

**Table 3:** Factors associated with poor ovarian response in women starting their first cycle of treatment.

	**Poor ovarian response^*1*^ N (%) (N=121,704 women)**	**Univariable risk ratio of poor ovarian response (99% CI)**	**P value**	**Multivariable^2^ risk ratio of poor ovarian response (99% CI) (N=108,857 women included in analysis)**	**P value^*3*^**
**All women**	**20, 621 (16.9%)**			**18, 240 (16.8%)**	
**Women’s age (years)**			P<0.001		P<0.001
18-34	7533 (11.8%)	1.0		1.0	
35-37	4832 (16.7%)	1.42 (1.35, 1.48)		1.38 (1.32, 1.44)	
38-39	3406 (22.9%)	1.94 (1.85, 2.03)		1.87 (1.78, 1.97)	
40-42	3442 (30.9%)	2.61 (2.49, 2.73)		2.53 (2.41, 2.66)	
43-44	1018 (41.8%)	3.53 (3.30, 3.78)		3.39 (3.15, 3.65)	
45-50	390 (51.0%)	4.31 (3.92, 4.75)		3.95 (3.50, 4.40)	
**Duration (years)**			P<0.001		P<0.001
<1	282 (14.0%)	0.86 (0.74, 0.99)		0.81 (0.70, 0.94)	
1-3	7253 (15.8%)	0.97 (0.93, 1.01)		0.93 (0.90, 0.97)	
4-6	6192 (16.3%)	1.0		1.0	
7-9	2311 (18.0%)	1.11 (1.05, 1.17)		1.12 (1.06, 1.18)	
10-12	1308 (19.4%)	1.19 (1.11, 1.28)		1.16 (1.08, 1.24)	
>12	1465 (22.4%)	1.38 (1.29, 1.47)		1.19 (1.11, 1.27)	
**Cause of infertility**			P<0.001		P<0.001
Tubal disease only	3300 (16.4%)	0.96 (0.91, 1.01)		1.00 (0.95, 1.06)	
Ovulatory only	1945 (22.2%)	1.29 (1.22, 1.37)		1.26 (1.18, 1.34)	
Male Factor Only	5599 (15.0%)	0.87 (0.83, 0.91)		1.98 (1.88, 2.09)	
Unexplained	5594(17.1%)	1.0		1.0	
Endometriosis	757 (18.5%)	1.08 (0.99, 1.18)		1.16 (1.05, 1.27)	
Cervical Factors only	12 (23.1%)	1.35 (0.70, 2.59)		1.21 (0.6, 2.45)	
Combination of Known factors	2586 (18.1%)	1.06 (1.00, 1.12)		1.63 (1.54, 1.73)	
**Previous pregnancy**			P<0.001		P<0.001
Yes	8800 (17.6%)	1.0		1.0	
No	11821 (16.5%)	0.94 (0.91, 0.97)		1.14 (1.10, 1.18)	
**Treatment**			P<0.001		P<0.001
IVF	16214 (22.4%)	1.0		1.0	
ICSI	4407 (8.9%)	0.40 (0.38, 0.42)		0.29 (0.27, 0.30)	
**Stimulated cycle**			P<0.001		
Yes	20094 (16.6%)				
No	527 (93.3%)				

^1^ Poor ovarian response defined as 3 or less oocytes retrieved. In 40 women information on number of oocytes retrieved was missing.

^2^ Risk ratio adjusted for all variables listed (except for stimulated cycle where 99% of women included in multivariate model had stimulated cycle).

^3^ P values obtained from multivariable model.

### Fertilisation in women who had at least one oocyte mixed with partner sperm

Of all women who started their first IVF cycle, 62,239 (86.0%) had at least one egg inseminated and of all women who started their first ICSI cycle, 49,119 (99.6%) had at least one egg injected with sperm. Of these, in the IVF group 2,931 (4.7%) women had failed fertilisation, while the corresponding figure in the ICSI group was 1,087 (2.2%). Poor fertilisation (1-19% eggs fertilised) occurred in 2.5% of IVF cycles and 0.9% of ICSI cycles. Table 4 shows univariable and multivariable associations with failure or poor fertilisation (<20% of eggs fertilised) stratified by type of treatment. In women undergoing IVF in their first cycle with at least one oocyte mixed with partner sperm, the risk of failure or poor fertilisation increased with increasing duration, for women who had no previous pregnancy (1.3, 99% CI 1.19, 1.42) and few (1-4) oocytes retrieved (RR 2.03, 99% CI 1.81, 2.27). A diagnosis of male factor increased the risk of failed or poor fertilisation whilst diagnosis of endometriosis or tubal disease reduced the risk when compared to women with unexplained infertility. In women who received ICSI, failed or poor fertilisation was associated with retrieval of few (1-4) oocytes (RR 5.29, 99% CI 4.32, 6.51) but not duration or cause of infertility. 

**Table 4:** Factors associated with poor fertilisation in women who had at least one oocyte mixed or injected with partner sperm.

	**IVF**			**ICSI**		
	**Univariable risk ratio of poor fertilisation^*1*^**	**Multivariable^2^ risk ratio (99% CI**)** of poor fertilisation**	**P value^*3*^**	**Univariable risk ratio of poor fertilisation^*1*^**	**Multivariable^2^ risk ratio (99% CI**)** of poor fertilisation**	**P value^*3*^**
	**(N=62,239 women)**	**(N=55, 725 women included in the analysis)**		**(N=49, 119 women)**	**(N=44,043 women included in analysis)**	
**All women**	**4500 (7.2%)**	**4006 (7.2%)**		**1518 (3.1%)**	**1321 (3.0%)**	
**Women’s age (years)**			P=0.024			P=0.001
18-34	1.0	1.0		1.0	1.0	
35-37	1.00 (0.91, 1.09)	0.93 (0.85, 1.03)		1.16 (0.98, 1.38)	0.96 (0.80, 1.15)	
38-39	1.02 (0.90, 1.14)	0.90 (0.79, 1.02)		1.64 (1.35, 1.99)	1.19 (0.96, 1.48)	
40-42	1.11 (0.98, 1.26)	0.93 (0.81, 1.07)		2.07 (1.69, 2.54)	1.27 (1.01, 1.59)	
43-44	1.33 (1.05, 1.68)	0.99 (0.76, 1.28)		2.83 (2.00, 4.00)	1.45 (0.99, 2.11)	
45-50	1.96 (1.41, 2.74)	1.35 (0.94, 1.95)		3.79 (2.25, 6.37)	1.65 (0.94, 2.90)	
**Duration (years)**			P<0.001			P=0.035
<1	0.78 (0.56, 1.07)	0.95 (0.68, 1.31)		0.88 (0.49, 1.58)	0.95 (0.54, 1.68)	
1-3	0.87 (0.79. 0.95)	0.90 (0.82, 0.99)		0.95 (0.81, 1.12)	0.96 (0.81, 1.13)	
4-6	1.0	1.0		1.0	1.0	
7-9	1.15 (1.01, 1.30)	1.24 (1.09, 1.40)		1.12 (0.89, 1.41)	1.09 (0.87, 1.38)	
10-12	1.03 (0.87, 1.22)	1.19 (1.00, 1.41)		1.42 (1.09, 1.85)	1.35 (1.03, 1.77)	
>12	0.99 (0.83, 1.17)	1.19 (0.99, 1.43)		1.21 (0.91, 1.62)	1.06 (0.78, 1.44)	
**Cause of infertility**			P<0.001			P=0.224
Tubal disease only	0.60 (0.54, 0.67)	0.64 (0.57, 0.71)		1.22 (0.85, 1.75)	1.26 (0.86, 1.85)	
Ovulatory only	1.09 (0.97, 1.23)	1.07 (0.94, 1.21)		1.57 (1.07, 2.30)	1.43 (0.96, 2.13)	
Male Factor Only	1.29 (1.14, 1.47)	1.29 (1.13, 1.48)		1.08 (0.87, 1.34)	1.16 (0.93, 1.45)	
Unexplained	1.0	1.0		1.0	1.0	
Endometriosis	0.75 (0.62, 0.91)	0.71 (0.58, 0.86)		1.08 (0.54, 2.14)	0.92 (0.43, 1.98)	
Cervical Factors only	1.78 (0.67, 4.73)	1.58 (0.55, 4.48)		-**^*4*^**	***^-4^***	
Combination of Known factors	0.93 (0.81, 1.07)	0.94 (0.81, 1.08)		1.14 (0.89, 1.47)	1.17(0.90, 1.52)	
**Previous pregnancy**			P<0.001			P=0.006
Yes	1.0	1.0		1.0	1.0	
No	1.33 (1.23, 1.43)	1.30 (1.19, 1.42)		0.99 (0.86, 1.13)	1.19 (1.01, 1.39)	
**Number of oocytes retrieved**			P<0.001			P<0.001
1-4	2.03 (1.83, 2.26)	2.03 (1.81, 2.27)		5.85 (4.83, 7.09)	5.29 (4.31, 6.51)	
5-9	1.10 (0.99, 1.22)	1.09 (0.98, 1.21)		1.49 (1.21, 1.83)	1.40 (1.13, 1.75)	
10-14	1.0	1.0		1.0	1.0	
15-19	0.95 (0.82, 1.09)	0.94 (0.81, 1.09)		0.76 (0.56,1.05)	0.73 (0.54, 1.02)	
20+	1.10 (0.95, 1.28)	1.08 (0.92, 1.27)		0.81 (0.56, 1.16)	0.82 (0.56, 1.19)	

^1^ Poor fertilisation defined as failure or less than 20% of oocytes fertilised

^2^ Multivariable risk ratio is adjusted for all variables listed

^3^ P values obtained from multivariable model.

^4^ Women receiving ICSI with cause of infertility recorded as cervical factors only (n=4), were excluded from the analysis

### Positive pregnancy test in women who had at least one embryo transferred

Of women undergoing their first cycle of IVF and ICSI and having at least one embryo created, 4.9% (2882) and 4.3% (2081) of women respectively, failed to achieve embryo transfer. In women who received one or more embryos, similar proportions of those treated by IVF and ICSI failed to achieve a positive pregnancy test, 33, 839 (60%) and 27, 317 (59.4%) respectively. 

Multivariable models showed the risk of a negative pregnancy test 2 weeks after embryo transfer in women who reached this stage, (using either IVF or ICSI generated embryos) increased with rising female age. Absence of a pregnancy was less likely in women with a short duration of infertility, but more likely in women with no previous pregnancy, low oocyte yield, poor embryo utilisation (<50%) and transfer of 1, 3 or 4 embryos (as opposed to 2) (Table 5). 

**Table 5:** Factors associated with failure to achieve positive pregnancy test in women who reached embryo transfer.

	**IVF**			**ICSI**		
**Characteristic**	**Univariable risk ratio of non-positive pregnancy test (99%CI)**	**Multivariable risk ratio^1^ of non-positive pregnancy test (99% CI)**	**P value^2^**	**Univariable risk ratio of non-positive pregnancy test (99%CI)**	**Multivariable risk ratio^1^ of non-positive pregnancy test (99% CI)**	**P value^2^**
	**N=56, 426 women with at least one embryo transferred**	**(N=50,591 women included in analysis)**		**N=45,951 women with at least one embryo transferred**	**(N=41, 310 women included in the analysis**	
**All Women**	**33,839 (60.0%)**	**30,336 (60.0%)**		**27,317 (59.4%)**	**24,547 (59.4%)**	
**Women’s age (years)**			P <0.001			P<0.001
18-34	1.0	1.0		1.0	1.0	
35-37	1.10 (1.08, 1.13)	1.09 (1.06, 1.11)		1.10 (1.07, 1.13)	1.06 (1.05, 1.09)	
38-39	1.21 (1.18, 1.24)	1.17 (1.14, 1.21)		1.22 (1.18, 1.26)	1.17 (1.13, 1.21)	
40-42	1.40 (1.37, 1.44)	1.33 (1.30, 1.37)		1.41 (1.37, 1.45)	1.30 (1.26, 1.49)	
43-44	1.64 (1.59, 1.69)	1.53 (1.48, 1.59)		1.60 (1,54, 1.67)	1.42 (1.36, 1.49)	
45-50	1.71 (1.64, 1.78)	1.55 (1.47, 1.63)		1.70 (1.62, 1.79)	1.49 (1.40, 1.59)	
**Duration (years)**			P<0.001			P<0.001
<1	0.78 (0.72, 0.86)	0.78 (0.71, 0.85)		0.80 (0.73, 0.89)	0.80 (0.72, 0.88)	
1-3	0.98 (0.96, 1.00)	0.96 (0.94, 0.98)		0.96 (0.93, 0.98)	0.95 (0.93, 0.97)	
4-6	1.0	1.0		1.0	1.0	
7-9	1.02 (0.99, 1.06)	1.01 (0.98, 1.04)		1.03 (0.99, 1.06)	1.02 (0.98, 1.05)	
10-12	1.04 (1.0, 1.08)	1.01 (0.97, 1.05)		1.05 (1.01, 1.10)	1.03 (0.98, 1.07)	
>12	1.09 (1.05, 1.14)	1.02 (0.98, 1.06)		1.13 (1.08, 1.17)	1.07 (1.02, 1.11)	
**Cause of infertility**			P<0.001			P=0.002
Tubal disease only	1.05 (1.03, 1.07)	1.10 (1.08, 1.13)		1.07 (1.02, 1.13)	1.09 (1.03, 1.15)	
Ovulatory only	0.98 (0.95, 1.01)	0.98 (0.95, 1.02)		1.02 (0.96, 1.09)	1.00 (0.94, 1.07)	
Male Factor Only	0.95 (0.92, 1.0)	0.98 (0.95, 1.02)		0.98 (0.95, 1.01)	1.01 (0.98, 1.05)	
Unexplained	1.0	1.0		1.0	1.0	
Endometriosis	0.98 (0.94, 1.02)	1.01 (0.67, 1.05)		0.96 (0.86, 1.07)	0.96 (0.86, 1.07)	
Cervical Factors only	1.23 (0.93, 1.64)	1.24 (0.91, 1.70)		-^4^	-^4^	
Combination of known factors	1.01 (0.98, 1.05)	1.05 (1.01, 1.09)		1.00 (0.96, 1.04)	1.01 (0.97, 1.05)	
**Previous pregnancy**			P<0.001			P<0.001
Yes	1.0	1.0		1.0	1.0	
No	1.01 (0.99, 1.02)	1.05 (1.03, 1.08)		0.99 (0.97, 1.01)	1.05 (1.03, 1.08)	
**Number of embryos transferred**			P<0.001			P<0.001
One	1.39 (1.36, 1.41)	1.26 (1.23, 1.29)		1.41 (1.38, 1.44)	1.26 (1.22, 1.29)	
Two	1.0	1.0		1.0	1.0	
Three/four	1.18 (1.15, 1.22)	1.06 (1.03, 1.10)		1.16 (1.12, 1.20)	1.05 (1.01, 1.09)	
**Number of oocytes retrieved**			P<0.001			P<0.001
1-4	1.35 (1.31, 1.38)	1.20 (1.16, 1.23)		1.38 (1.34, 1.42)	1.21 (1.17, 1.25)	
5-9	1.13 (1.11, 1.16)	1.11 (1.08, 1.14)		1.15 (1.12, 1.18)	1.11 (1.08, 1.14)	
10-14	1.0	1.0		1.0	1.0	
15-19	0.96 (0.93, 1.0)	0.98 (0.94, 1.01)		0.95 (0.92, 0.99)	0.96 (0.93, 1.00)	
20+	0.94 (0.90, 0.98)	0.96 (0.92, 1.01)		0.92 (0.88, 0.97)	0.94 (0.90, 0.99)	
**Embryo Utilisation^*3*^**			P<0.001			P=0.002
0-25%	0.90 (0.88,0.93)	1.05 (1.02, 1.09)		0.86 (0.83, 0.89)	1.02 (0.98, 1.06)	
25-50%	0.95 (0.93, 0.97)	1.01 (0.99, 1.04)		0.94 (0.92, 0.99)	1.03 (1.00, 1.06)	
50-75%	0.91 (0.89, 0.93)	0.98 (0.96, 1.01)		0.90 (0.88, 0.93)	0.99 (0.96, 1.02)	
75-100%	1.0	1.0		1.0	1.0	

^1^ Multivariable risk ratio is adjusted for all variables listed

^2^ P values obtained from multivariable model.

***^3^*** Embryo utilisation is defined as (number of embryos transferred + number of embryos frozen)/total number of embryos created

^4^ Women receiving ICSI with cause of infertility recorded as cervical factors only (n=4), were excluded from the analysis

### Live birth and singleton term live birth in those with a positive pregnancy test

A total of 41,221 women had positive pregnancy test. Of these, 11,466 (27.8%) failed to achieve a live birth and 21,421 (52.0%) failed to achieve a term, singleton live birth (Table 6). In women who became pregnant, increasing age, diagnosis of tubal disease, single embryo transfer and low number of oocytes retrieved were associated with lack of live birth. Inability to have a singleton livebirth was associated with age above 40 years, and transfer of 2 or more embryos. Number of oocytes retrieved, embryo utilisation and treatment by IVF or ICSI were not associated with lack of term singleton live birth. 

**Table 6:** Factors associated with non-live birth and non-term singleton live birth in women who achieved a pregnancy.

	**Non-live birth**			**Non-singleton term live birth^1^**		
	**Univariable risk Ratio of non-live birth**	**Multivariable^2^ risk Ratio of non-live birth**	**P value^3^**	**Univariable risk Ratio of non-term singleton live birth**	**Multivariable^2^ risk Ratio of non-term singleton live birth**	**P Value^3^**
	**(N=41,221 women)**	**(N= 37,018 women included in analysis)**		**(N=41,221 women)**	**(n=37,018 women included in analysis)**	
**All women**	**11,466 (27.8%)**	**10,276 (27.8%)**		**21,421 (52.0%)**	**19,252 (52.0%)**	
**Women’s age (years)**			P<0.001			P<0.001
18-34	1.0	1.0		1.0	1.0	
35-37	1.15 (1.09, 1.21)	1.14 (1.08, 1.21)		0.96 (0.93, 0.99)	0.96 (0.93, 0.99)	
38-39	1.43 (1.34, 1.52)	1.43 (1.34, 1.52)		0.98 (0.94, 1.02)	0.98 (0.94, 1.02)	
40-42	1.91 (1.79, 2.04)	1.83 (1.69, 1.97)		1.11 (1.05, 1.16)	1.07 (1.01, 1.14)	
43-44	2.68 (2.35, 3.06)	2.39 (2.04, 2.80)		1.39 (1.24, 1.55)	1.29 (1.13, 1.48)	
45-50	2.58 (1.87, 3.55)	2.50 (1.83, 3.42)		1.56 (1.28, 1.91)	1.46 (1.14, 1.87)	
**Duration (years)**			P<0.001			P<0.001
<1	1.06 (0.92, 1.22)	1.02 (0.88, 1.17)		0.95 (0.87, 1.04)	0.94 (0.86, 1.03)	
1-3	0.98 (0.93, 1.03)	0.95 (0.90, 1.00)		0.97 (0.94, 1.0)	0.96 (0.94, 0.99)	
4-6	1.0	1.0		1.0	1.0	
7-9	1.10 (1.02, 1.18)	1.06 (0.99, 1.14)		1.00 (0.96, 1.05)	1.00 (0.95, 1.04)	
10-12	1.15 (1.05, 1.26)	1.08 (0.99, 1.19)		1.03 (0.98, 1.09)	1.01 (0.96, 1.07)	
>12	1.24 (1.13, 1.36)	1.09 (0.98, 1.20)		1.07 (1.01, 1.14)	1.05 (0.99, 1.12)	
**Cause of infertility**			P<0.001			P<0.001
Tubal disease only	1.09 (1.03, 1.16)	1.15 (1.08, 1.23)		1.06 (1.03, 1.10)	1.05 (1.01, 1.09)	
Ovulatory only	1.02 (0.94, 1.11)	1.07 (0.98,1.17)		1.06 (1.00, 1.11)	1.05 (1.00, 1.11)	
Male Factor Only	0.94 (0.89, 0.99)	0.98 (0.91, 1.05)		0.96 (0.93, 1.00)	0.97 (0.93, 1.01)	
Unexplained	1.0	1.0		1.0	1.0	
Endometriosis	0.92 (0.82, 1.04)	0.97 (0.86, 1.10)		1.01 (0.94, 1.08)	1.01 (0.94, 1.09)	
Cervical Factors only	1.34 (0.41, 4.36)	1.49 (0.45, 4.96)		1.45 (0.86, 2.46)	1.52 (0.93, 2.50)	
Combination of Known factors	1.03 (0.96, 1.11)	1.08 (1.00, 1.16)		1.04 (1.0, 1.09)	1.04 (1.00, 1.09)	
**Previous pregnancy**			P=0.427			P=0.007
Yes	1.0	1.0		1.0	1.0	
No	0.90 (0.97, 0.94)	0.99 (0.94, 1.03)		0.96 (0.94, 0.99)	0.97 (0.94, 1.00)	
**Number of embryos transferred**			P<0.001			P<0.001
One	1.24 (1.14, 1.35)	1.17 (1.07, 1.28)		0.78 (0.72, 0.83)	0.79 (0.73, 0.85)	
Two	1.0	1.0		1.0	1.0	
Three/four	1.29 (1.20, 1.39)	1.06 (0.98, 1.15)		1.15 (1.10, 1.20)	1.11 (1.06, 1.17)	
**Number of oocytes retrieved**			P<0.001			P=0.227
1-4 oocytes	1.29 (1.20, 1.39)	1.20 (1.11, 1.30)		0.95 (0.90, 1.0)	0.98 (0.93, 1.04)	
5-9 oocytes	1.13 (1.08, 1.19)	1.11 (1.05, 1.17)		1.0 (0.97, 1.03)	1.00 (0.96, 1.03)	
10-14 oocytes	1.0	1.0		1.0	1.0	
15-19 oocytes	1.02 (0.95, 1.09)	1.06 (0.99, 1.13)		1.01 (0.98, 1.05)	1.02 (0.98, 1.08)	
20+ oocytes	0.98 (0.91, 1.06)	1.01 (0.93, 1.10)		1.03 (0.98, 1.07)	1.03 (0.98, 1.08)	
**Treatment**			P=0.128			P=0.572
IVF	1.0	1.0		1.0	1.0	
ICSI	0.96 (0.92, 1.00)	1.04 (0.98, 1.10)		0.96 (0.94, 0.99)	0.99 (0.96, 1.03)	
**Embryo Utilisation^*4*^**			P=0.061			P=0.940
0-25%	0.98 (0.91, 1.04)	1.05 (0.98, 1.13)		1.01 (0.97, 1.05)	0.99 (0.95, 1.04)	
25-50%	1.03 (0.98, 1.08)	1.05 (0.99, 1.11)		1.01 (0.98, 1.05)	1.00 (0.97, 1.03)	
50-75%	0.99 (0.94, 1.05)	1.00 (0.94, 1.07)		1.03 (1.0, 1.07)	1.00 (0.97, 1.04)	
75-100%	1.0	1.0		1.0	1.0	

^1^ Term singleton live birth was defined as a single baby born at 37 weeks or greater gestation

^2^ Multivariable risk ratio is adjusted for all variables listed in table

^3^ P values obtained from multivariable model

***^4^*** Embryo utilisation was defined as (number of embryos transferred + number of embryos frozen)/total number of embryos created

## Discussion

### Principal findings

The results of this study underline the importance of increased female age and duration of infertility, lack of previous pregnancy and tubal and male factor infertility in predicting the risk of not having a livebirth in an IVF treatment. At each stage, a woman’s chance of proceeding to the next phase of IVF treatment is affected by increased age and duration of infertility. The intention to perform ICSI is associated with a decreased risk of treatment failure in women starting an IVF cycle but this association is reversed at a later stage once fertilisation has been confirmed. Other factors such as cause of infertility, number of oocytes retrieved and number of embryos replaced also play a role in determining poor intermediate outcomes. 

### Strengths

This is the first study to conduct an analysis based on women as opposed to cycles using a large national IVF database with total capture of all treatments. It is also the first to attempt to identify factors affecting global failure as well as those influencing women’s chances of progressing onto the next stage of treatment. In recognition of a healthy baby as a key outcome of IVF, the results include factors which affect the chance of not having a singleton term livebirth.

### Weaknesses

As the study is based on routinely collected data, we were not able to adjust for a number of known and potential confounders such as smoking, body mass index and paternal age which are not captured at all within this dataset. In addition we were unable to examine the influence of tests of ovarian reserve or embryo quality as these data are not collected, although we did attempt to examine embryo quality through our derived variable embryo utilisation. To maintain confidentiality, maternal age was only available grouped into categories – this inevitably impacted on the accuracy of the model to estimate the effect of increasing age on treatment outcomes. There were some missing data for some of the variables. In order to analyse outcomes in women as opposed to cycles we focused on first cycles only and are therefore unable to identify factors associated with live births resulting from multiple IVF cycles including fresh and frozen embryo transfers. 

### Comparison with results in the published literature

A number of previous studies (including two based on the HFEA database) have estimated factors affecting outcomes in IVF[15,18,25]. All have used a cycle based approach and as the data are not linked to women have been unable to adjust for the clustering effect of cycles within women. In their analysis of 36, 961 in-vitro fertilisation (IVF) cycles between 1991 and 1994 Templeton et al. found that livebirth rates were highest in women aged 25-30 years, with poorer outcomes in older women[18]. Age adjusted livebirth rates fell with increasing duration of infertility, but the cause of infertility had little effect on outcomes. The odds of success were enhanced by previous pregnancy and livebirth and reduced by one or more previous failed IVF treatment cycles reduced them. 

A more recent study, based on the analysis of HFEA data between 2003 and 2007, examined the predictors of live birth in 144,018 IVF and intracytoplasmic sperm injection (ICSI) cycles[15]. The overall livebirth rate in this cohort was 23.4% (95% confidence intervals 23.2-23.7). The odds of live birth following IVF/ICSI were increased by lower female age, shorter duration of infertility, previous successful IVF treatments, use of donor oocytes and unexplained infertility. Women who underwent ICSI had a higher chance of a livebirth compared to those who had IVF. However, our analyses suggest that in women who have had oocyte retrieval, treatment by ICSI does not appear to offer increased benefit and may be actually associated with failure to achieve live birth. This is likely to be due to the change in the composition of women who reach the stage of fertilisation. Many more women whose intention was to be treated by IVF (13.6%) did not have any oocytes retrieved compared to those women whose intended treatment was ICSI (0.3%). This is likely to be due to the fact that many more women who require IVF may be older and less likely to respond well to ovarian stimulation compared to women receiving ICSI. Many in this group are not themselves infertile but are relatively young partners of men with semen abnormalities.

Our results, based on the same dataset but using a different timeframe and adopting a woman based approach, corroborate the findings of these earlier studies in confirming the pivotal role of these predictive variables. 

A preliminary analysis of these data, to investigate the factors associated with failure to achieve pregnancy in women undergoing first IVF and ICSI cycles that reached embryo transfer [26] adopted a different analytical approach with odds ratios reported rather than risk ratios. In general, these odds ratios were substantially larger than the corresponding risk ratios reported in Table 5, due to the high proportion of women (60%) who failed to achieve pregnancy following embryo transfer. In these circumstances where the outcome is common, the odds ratio is known to overstate the risk ratio [24]. 

A systematic review of studies by Loendersloot[17] included a number of prognostic models but did not include the two studies mentioned above (Templeton and Nelson[15,18]) but identified others which have generated models for the prediction of pregnancy following IVF. The outcome in most of these studies was pregnancy[2-9,11] rather than livebirth[10,12,14], and the individual papers varied in the factors identified – possibly because they were based on routinely collected data and were therefore reliant on the variables available in the relevant dataset. Aggregation of the results showed negative associations between the chance of pregnancy and the following – female age, duration of infertility and basal FSH. It is worth noting that the association with age was stronger than that with duration (OR 0.99, [95% CI 0.98, 1.00]). Retrieval of an increased number of oocytes was associated with higher chances of pregnancy, a finding which has been reported by others[21,27].

Few large studies are able to report on the predictive power of embryo quality[7,9,12] and those that do, are inconsistent in the way embryos are graded. Hunault[7] identified the morphology score of the two best embryos available for transfer as a predictor of ongoing pregnancy following single embryo transfer.

Although the importance of these predictive factors has been known for some time, their relative importance at each stage of the treatment, in terms of predicting overall success as well as the chance of proceeding to the next stage has not been reported previously. This has been addressed in this study, and while the factors are the same it is interesting to see how their influence changes over the course of an IVF cycle. The association between ICSI and lower risk of treatment failure is particularly interesting. Previous work[15] has highlighted the positive association between live birth rates and the use of ICSI. The role of ICSI appears to be more complex than previously imagined, as seems to reflect the fact that women undergoing ICSI have similar prognosis to those undergoing IVF once women with no oocytes retrieved are no longer included in the analysis.

Our results suggest that increased female age is a key determinant of treatment failure - globally as well as at each stage. Though less pronounced, the influence of duration of infertility is present at all stages except in the prediction of fertilisation following insemination or injection of embryos. Lack of previous pregnancy also has a negative effect all along the course of treatment up to the point of confirmation of biochemical pregnancy. Tubal infertility with associated hydrosalpinx affects the chance of implantation of a transferred embryo – an observation that has been reported previously and has been considered to be due to the effect of secretions in the fallopian tube which may potentially flow into the endometrial cavity and impair the quality of the deciduas. This effect has clinical relevance as a Cochrane review has confirmed doubling of IVF related pregnancy rates after salpingectomy in women with hydrosalpinges[28].

### Clinical implications

The ability to predict chances of pregnancy and livebirth is critical to decision making around IVF. In cases where the outcome is in doubt, IVF itself is often seen as a prognostic exercise which is able to reveal which women are likely to respond to ovarian stimulation and produce good quality embryos. The ability to identify factors associated with failure at each stage of the treatment has the ability to refine decision making around proceeding with treatment – especially where the prognosis changes during treatment. For example a 40 year old woman with a poor oocyte yield is more likely to be unsuccessful than one with a better oocyte yield. This information can be quantified and can potentially lead to more sophisticated strategies for individualised decision making such that women can be counselled appropriately regarding risks of non pregnancy leading to live birth at each stage.

#### Research implications

This strategy opens up the possibilities of new and more nuanced methods of analyses of the U.K. national IVF dataset. The ability to link cycles within women offers an opportunity to develop woman based models in IVF – with better prediction of cumulative outcomes over a number of fresh and frozen IVF cycles.

## Conclusion

Female age is a key predictor of failure to have a livebirth following IVF as well as the risk of poor performance at each stage of treatment. While increased duration of infertility is also associated with worse outcomes at every stage, its impact appears to be less influential. Absence of a previous pregnancy did not impact on ovarian stimulation and pregnancy loss but does affect the chances of fertilisation and positive pregnancy test. Women embarking on ICSI treatment for male factor infertility have a lower chance of treatment failure but this does not appear to be due to increased chances of implantation of ICSI embryos.
